# Circular RNA in tumor metastasis

**DOI:** 10.1016/j.omtn.2021.01.032

**Published:** 2021-02-04

**Authors:** Chao Zhang, RongFang Ding, YiCheng Sun, Si Tong Huo, Alina He, Chang Wen, HongHao Chen, William W. Du, WeiNan Lai, Huijun Wang

**Affiliations:** 1 Department of Biochemistry and Molecular Biology, School of Basic Medical Science, Southern Medical University and Guangdong Provincial Key Laboratory of Single Cell Technology and Application, Guangzhou 510000, Guangdong Province, China; 2 Department of Laboratory Medicine, Nanhai Hospital, Southern Medical University, Foshan 510000, Guangdong Province, China; 3 Department of Pediatrics, Nanfang Hospital, Southern Medical University, Guangzhou 510000, Guangdong Province, China; 4 Department of Biochemistry, University of Toronto, Toronto, ON M5S 1A8, Canada; 5 Sunnybrook Research Institute, Sunnybrook Health Sciences Centre, Toronto, ON M4N 3M5, Canada; 6 Department of Laboratory Medicine and Pathobiology, University of Toronto, Toronto, ON M5S 1A1, Canada; 7 Department of Rheumatology, Nanfang Hospital, Southern Medical University, Guangzhou 510515, Guangdong Province, China 510515; 8 School of Medicine, Tsinghua University, Beijing 100084, China

**Keywords:** circular RNA, cancer, metastasis, gene regulation

## Abstract

Circular RNAs (circRNAs) are a type of endogenous non-coding RNA that were discovered to regulate gene expression through multiple pathways. Metastasis remains one of the biggest obstacles in cancer treatment. In this review, we focus on circRNAs involved in cancer tumorigenesis and metastasis. We present recently identified tumor-related circRNAs and discuss their functioning in tumor progression and metastasis. These circRNAs are categorized into different functional mechanisms, including microRNA (miRNA) sponging, protein binding, regulation of host genes, translation of circRNAs, and exosomal circRNAs. Additionally, the indirect functions of circRNAs that regulate epithelial-mesenchymal transition and autophagy are also discussed.

## Graphical Abstract

**Figure:**
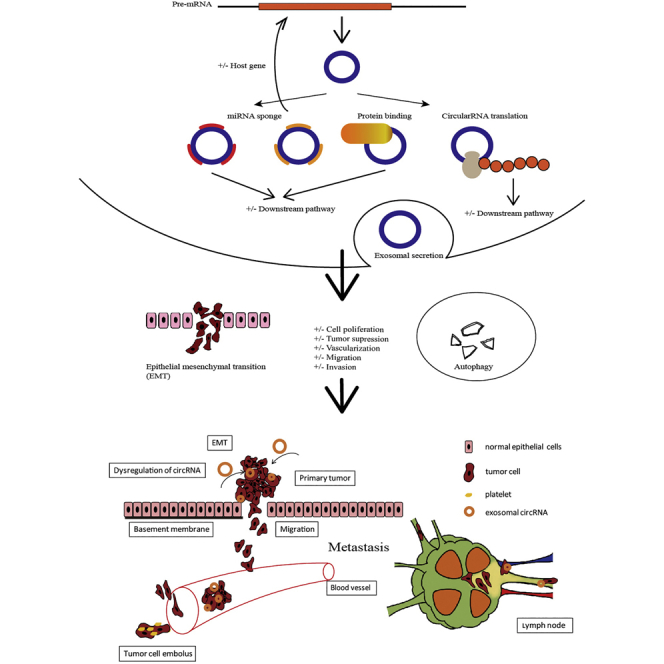

## Teaser

Large numbers of differentially expressed circRNAs have been identified in tumors and metastasis. Gain-of-function and loss-of-function approaches have been developed to manipulate specific circRNA expression in order to modulate tumor development. circRNAs may be used as potential biomarkers for early diagnosis and may be targets for therapeutic strategies.

## Main text

Circular RNAs (circRNAs) were first found in 1976,^1^ and the first circRNA derived from a candidate tumor suppressor gene was identified in 1991.^2^ Recently, an increasing number of discoveries in circRNA research have been made due to the development of bioinformatics technologies and next-generation RNA sequencing (RNA-seq).^3^, ^4^, ^5^, ^6^, ^7^ The total number of known circRNAs has accumulated to 30,000 in recent years. In recent years, emerging research discovered that circRNAs were involved in pathological processes, and it is possible that quantifiable circRNA expression could become biomarkers for some long-term diseases such as cancer and heart diseases. In this review, we summarize the recent studies of circRNAs in tumor metastasis studies and categorize the studies in terms of the circRNA functions (Table 1). This provides thorough coverage of current research status of circRNAs in metastasis. This would be inspirational since a number of circRNAs are common players in a variety of disease conditions.

**Table 1:** Summary of mentioned and supplementary circRNAs according to functions and cancer types

cRNA	Cancer type	Metastasis	Gene	Wathway	Expression (+/−)	Downstream effect	Mechanism	Manipulated expression to ameliorate disease	Reference
circRGNEF	bladder cancer	lymphatic metastasis	RGNEF	miR-548/KIF2C	+	reduces MMP2 levels via the circEHMT1/miR-1233-3p/KLF4 axis to inhibit metastasis	miRNA sponge	yes	^8^
circPRRC2A	renal cell carcinoma	lymphatic metastasis	PRRC2A	DHX9-circPRRC2A-miR-514a-5p/miR-6776-5p-TRPM3	+	miR-514a-5p/miR-6776-5p-TRPM3; TRPM3/SNAIL/vimentin; DHX9-circPRRC2A-miR-514a-5p/miR-6776-5p-TRPM3	miRNA sponge	yes	^9^
circAKT3	clear cell renal cell carcinoma	not mentioned	AKT3	circAKT3/miR-296-3p/E-cadherin axis	−	promotes metastasis of clear cell renal cell carcinoma by sponging miR-296-3p and upregulating E-cadherin expression	miRNA sponge, EMT	yes	^10^
circTLK1	renal cell carcinoma	not mentioned	TLK1	circTLK1/miR-136-5p/CBX4/VEGFA axis	+	sponges miR-136-5p to regulate circTLK1 expression, promoting the tumorigenesis and development of renal cell carcinoma	miRNA sponge	yes	^11^
circFGFR1	non-small cell lung cancer	lymphatic metastasis	FGFR1	circFGFR1/CXCR4	+	sponges miR-381-3p to upregulate the expression of CXCR4	miRNA sponge	yes	^12^
circRPMS1	naso pharyngeal carcinoma	not mentioned	RPMS1	not mentioned	+	sponges miR-203, miR-31, and miR-451 to inhibit nasopharyngeal carcinoma cell growth and EMT	miRNA sponge; EMT	yes	^13^
circCRIM1	lung adenocarcinoma	lymphatic metastasis	CRIM1	miR-182/miR-93-LIFR	−	promotes the expression of LIFR by sponging miR-93 and miR-182	miRNA sponge	yes	^14^
circCYFIP2	gastric cancer	lymphatic metastasis	CYFIP2	miR-1205/E2F1 axis	+	promotes gastric cancer cell growth and motility through sponging miR-1205 to upregulate E2F1	miRNA sponge	yes	^15^
circRUNX1	colorectal cancer	not mentioned	RUNX1	miR-145-5p/IGF1	+	acts as a ceRNA for miR-145-5p to upregulate IGF1	miRNA sponge	yes	^16^
circLMTK2	gastric cancer	lymphatic metastasis	LMTK2	miR-150-5p/c-Myc axis	+	sponges miR-150-5p to upregulate c-Myc	miRNA sponge	yes	^17^
hsa_circRNA_0023404	cervical cancer	lymphatic metastasis	RNF121	miR-5047/VEGFA and autophagy signaling	+	sponges miR-5047 to regulate VEGFA and autophagy signaling	miRNA sponge	yes	^18^
circEHMT1	breast cancer	lymphatic metastasis	EHMT1	miR-1233-3p/KLF4/MMP2 axis	−	sponges miR-1233-3p to downregulate KLF4 and reduce MMP2 level	miRNA sponge	yes	^19^
hsa_circRNA_0032821	gastric cancer	lymphatic metastasis	CEP128	MEK1/ERK1/2	+	acts as an oncogene in gastric cancer cell proliferation, EMT, autophagy, migration and invasion *in vitro*, and tumor growth *in vivo* by activating the MEK1/ERK1/2 axis	protein binding	yes	^20^
circGSK3β	esophageal squamous cell carcinoma	lymphatic metastasis	GSK3β	GSK3β/β-catenin	+	interacts with GSK3β to promote the activity of β-catenin in esophageal squamous cell carcinoma cells	protein binding; regulate host gene	yes	^21^
circNSUN2	colorectal carcinoma	lymphatic metastasis	NSUN2	HMGA2	+	forms the circNSUN2/ IGF2BP2/HMGA2 RNA-protein ternary complex and promotes lymphatic metastasis of colorectal carcinoma through the HMGA2 pathway	protein binding	yes	^22^
circHuR	gastric cancer	lymphatic metastasis	HuR	not mentioned	−	restrains the transcription of HuR by inhibiting CNBP transactivation through interacting with its RGG domain	protein binding; regulates host gene	yes	^23^
circFOXK2	pancreatic ductal adenocarcinoma	not mentioned	FOXK2	not mentioned	+	sponges miR-942 to promote the expressions of ANK1, GDNF, and PAX6; interacts with YBX1 and hnRNPK to promote the expressions of oncogenic proteins NUF2 and PDXK	protein binding; miRNA sponge	yes	^24^
circASAP1	hepatocellular carcinoma	not mentioned	ASAP1	miR-326/miR-532-5p-MAPK1/CSF-1	+	decreases miR-326 and miR-532-5p level, thus promoting proliferation and invasion of HCC through miR-326/MAPK/ERK1/2 signaling and raising TAM infiltration through CSF-1 activation and secretion	miRNA sponge	yes	^25^
circCCNB1	breast cancer	not mentioned	CCNB1	not mentioned	-	forming a complex with H2AX and Bclaf1 to induce cell death in p53 mutant cells	protein binding; apoptosis	yes	^26^
circCCNB1	not mentioned	not mentioned	CCNB1	not mentioned	−	forms a complex containing circ-Ccnb1, Ccnb1, and Cdk1 to inhibit the oncogenic function of Ccnb1	protein binding	yes	^27^
circSKA3	breast cancer	not mentioned	SAK3	not mentioned	+	complexes with Tks5 and integrin β1, inducing invadopodium formation	protein binding	yes	^28^
circSEPT9	triple-negative breast cancer	lymphatic metastasis	SEPT9	LIF/Stat3	+	medicated by E2F1 and EIF4A3 and then sponges miR-637 and activates the LIF/Stat3 signaling pathway	protein binding; miRNA sponge	yes	^29^
circENO1	lung adenocarcinoma	not mentioned	ENO1	circ-ENO1/miR-22-3p/ENO1	+	sponges miR-22-3p to upregulate its host gene and promote metastasis	regulates its host gene; miRNA sponge	yes	^30^
circITGA7	colorectal cancer	lymphatic metastasis	ITGA7	RAS	−	suppresses the Ras signaling pathway by competitively binding to miR-370-3p to upregulate NF1 translation; upregulates the transcription of its host gene ITGA7 by suppressing RREB1 via the Ras pathway	regulate its host gene; miRNA sponge	yes	^31^
circYAP	breast carcinoma	not mentioned	YAP	not mentioned	+	abolishes the interaction of PABP on the poly(A) tail with eIF4G on the 5′-cap of the Yap mRNA and suppress its translation	regulates its host gene; protein binding	yes	^32^
hsa_circRNA_100338	hepatocellular carcinoma	not mentioned		not mentioned	+	participates in the regulation of angiogenesis and HCC metastasis	Exosome	yes	^33^
circABCC1	colorectal cancer	not mentioned	ABCC1	Wnt; Wnt/β-catenin	+	exosomal circABCC1 from CD133^+^ cells mediates cell stemness and metastasis in colorectal cancer through WNT signaling	Exosome	yes	^34^
hsa_circRNA_044516	prostate cancer	not mentioned	COL1A1	not mentioned	+	sponges miR-29a-3p to promote prostate cancer metastasis	exosome; miRNA sponge	yes	^35^
circMMP2	hepatocellular carcinoma	not mentioned	MMP2	circ_MMP2/miR-136-5p/MMP2	+	regulates its host gene through sponging sequestering miR-136-5p	exosome; miRNA sponge; regulates host gene	yes	^36^
hsa_circ_0051443	hepatocellular carcinoma	not mentioned	TRAPPC6A	not mentioned	−	released by normal cells and transported from normal cells to HCC cells to inhibit malignant phenotype by sponging miR-331-3p to protect BAK1	exosome; miRNA sponge	yes	^37^
hsa_circ_0001165	prostate cancer	not mentioned	NCOA3	not mentioned	+	regulates TNF expression through hsa_x0002_miR-187-3p to induce EMT	EMT; miRNA sponge	not mentioned	^38^
hsa_circ_0001085	prostate cancer	not mentioned	GLS	PI3K-Akt, TGF-β, MAPK	−	indirectly regulates several pathways to inhibit EMT	EMT	not mentioned	^38^
circSCYL2	breast cancer	not mentioned	SCYL2	OSR1/TGF-β	−	may regulate EMT through OSR1/TGF-β axis	EMT	yes	^39^
hsa_circ_0023642	gastric cancer	not mentioned	UVRAG	not mentioned	+	promotes EMT, functions as a tumor promoter in gastric cancer	EMT	yes	^40^
circHIPK3	cervical cancer	not mentioned	HIPK3	circ-HIPK3/miR-338-3p/HIF-1α	+	sponges miR-338-3p to up_x0002_regulate the HIF-1α expression, and contributes to the cervical cancer cell EMT	EMT; miRNA sponge	yes	^41^
circPTK2	non-small cell lung cancer	lymphatic metastasis	PTK2	TGF-β/Smad	−	protects TIF1γ by sponging out miR-429/miR-200b-3p to inhibit EMT	EMT; miRNA sponge	yes	^42^
circPTPRA	non-small cell lung cancer	lymphatic metastasis	PTPRA	circPTPRA/miR-96-5p/ RASSF8/E cadherin	−	sponges miR-96-5p to upregulate the downstream tumor suppressor RASSF8	EMT; miRNA sponge	yes	^43^
hsa_circ_0006948	esophageal squamous cell carcinoma	lymphatic metastasis	FNDC3B	hsa_circ_0006948/miR-490-3p/HMGA2	+	upregulates HMGA2 and induces EMT by sponging miR-490-3p	EMT; miRNA sponge	yes	^44^
circDNMT1	breast cancer	not mentioned	DNMT1	not mentioned	+	interacts with p53 and AUF1 proteins to promote their nuclear translocation	autophagy; protein binding	yes	^45^
circMUC16	epithelial ovarian cancer	not mentioned	MUC16	circMUC16, RUNX1, Beclin1, ATG13, and miR-199a	+	mediated autophagy accelerates the malignant behavior of epithelial ovarian cancer	autophagy	yes	^46^
hsa_circRNA_0067934	hepatocellular carcinoma	hematogenous metastasis	PRKCI	miR-1324/FZD5/Wnt/β-catenin axis	+	directly suppresses miR-1324, which can downregulate the Wnt/β-catenin signaling pathway in HCC	miRNA sponge	not mentioned	^47^
circHIPK3	colorectal cancer	hematogenous metastasis	HIPK3	c-Myb/circHIPK3/miR-7 axis	+	serves as a novel oncogenic circRNA by sponging miR-7	miRNA sponge	yes	^48^
circPVT1	colorectal cancer	hematogenous metastasis	PVT1	circPVT1/miR-145 axial	+	works as an oncogene, promotes metastasis via miR-145 sponging in CRC	miRNA sponge	yes	^49^
circHIPK3	osteosarcoma	hematogenous metastasis	HIPK3	miR-637/STAT3 Axis	+	promotes STAT3 expression via interacting with miR-637 in OS cells	miRNA sponge	yes	^50^
circHIPK3	bladder cancer	lymphatic metastasis	HIPK3	miR-558/heparanase axis	−	inhibits aggressiveness and metastasis of bladder cancer cells through targeting miR-558/heparanase axis	miRNA sponge	Yes	^51^
circITCH	bladder cancer	lymphatic metastasis	ITCH	circ-ITCH/miR-17, miR-224/p21, PTEN	−	sponges miR-17 and miR-224 to suppress cell proliferation, migration, and invasion	miRNA sponge	yes	^52^

ABCC1, ATP binding cassette subfamily C member 1; AKT3, AKT serine/threonine kinase 3; ASAP1, microtubule-associated protein 1; AUF1, AU-rich element-binding factor 1; Bclaf1, B cell leukemia/lymphoma 2 apoptosis regulator-associated transcription factor 1; CBX4, chromobox 4; ceRNA, competing endogenous RNA; CCNB1, cyclin B1; Cdk1, cyclin-dependent kinase 1; circABCC1, circRNA ATP binding cassette subfamily C member 1; circAKT3, circRNA AKT serine/threonine kinase 3; circCNNB1, circRNA cyclin B1; circCRIM1, circRNA cysteine-rich transmembrane BMP regulator 1; circCYFIP2, circRNA cytoplasmic FMR1 interacting protein 2; circDNMT1, circRNA DNA methyltransferase 1; circEHMT1, circRNA euchromatic histone-lysine-N-methyltransferases 1; circENO1, circRNA enolase 1; circFGFR1, circRNA fibroblast growth factor receptor 1; circFOXK2, circRNA forkhead box K2; circGSK3β, circRNA glycogen synthase kinase-3β; circHIPK3, circRNA homeodomain interacting protein kinase 3; circHuR, circRNA human antigen R; circITCH, circRNA itchy E3 ubiquitin protein ligase; circITGA1, circRNA integrin subunit alpha 7; circLMTK2, circRNA lemur tyrosine kinase 2; circMMP2, circRNA matrix metallopeptidase 2; circMUC16, circRNA mucin 16, cell surface associated; circNSUN2, circRNA NOP2 RNA methyltransferase 2; circPTK2, circRNA protein tyrosine kinase 2; circPTPRA, circRNA protein tyrosine phosphatase receptor type A; circRGNEF, circRNA Rho guanine nucleotide exchange factor; circRPMS1, circRNA human gammaherpesvirus 4; circRUNX1, circRNA RUNX family transcription factor 1; circSCYL2, SCY1-like pseudokinase 2; circSEPT9, circRNA septin 9; circSKA3, circRNA spindle and kinetochore-associated complex subunit 3; circTLK1, circRNA tousled-like kinase 1; CNBP, CCHC-type zinc-finger nucleic acid binding protein; CRIM1, cysteine-rich transmembrane BMP regulator 1; CSF-1, colony-stimulating factor 1; CXCR4, C-X-C motif chemokine receptor 4; DNMT1, DNA methyltransferase 1; CYFIP2, cytoplasmic FMR1 interacting protein 2; E2F1, E2F transcription factor 1; EHMT1, euchromatic histone-lysine-N-methyltransferases 1; EIF4A3, eukaryotic translation initiation factor 4A3; eIF4G, eukaryotic translation initiation factor 4 gamma; ENO1, enolase 1; FGFR1, fibroblast growth factor receptor 1; FOXK2, forkhead box K2; H2AX, histone family 2A variant; HIPK3, homeodomain interacting protein kinase 3; HMGA2, high mobility group AT-hook 2; hnRNPK, heterogeneous nuclear ribonucleoprotein K; HuR, human antigen R; IGF1, insulin-like growth factor 1; ITCH, itchy E3 ubiquitin protein ligase; KIF2C, kinesin family member 2C; LMTK2, lemur tyrosine kinase 2; MAPK1, mitogen-activated protein kinase 1; MMP2, matrix metallopeptidase 2; MUC16, mucin 16, cell surface associated; NUF2, nuclear division cycle 80 kinetochore complex component; OSR1, odd-skipped related transcription factor 1; PABP, poly(A)-binding protein; PDXK, pyridoxal kinase; PTK2, protein tyrosine kinase 2; PTPRA, protein tyrosine phosphatase receptor type A; RGNEF, Rho guanine nucleotide exchange factor; RPMS1, human gammaherpesvirus 4; RREB1, Ras responsive element binding protein 1; RUNX1, RUNX family transcription factor 1; SCYL2, SCY1-like pseudokinase 2; SEPT9, septin 9; SKA3, spindle and kinetochore-associated complex subunit 3; TGF-β, transforming growth factor β; TIF1γ, transcriptional intermediary factor 1 gamma; Tks5, sperm hammerhead 3 and protamine 3 domains 2A; TLK1, tousled-like kinase 1; TNF, tumor necrosis factor; VEGFA, vascular endothelial growth factor A; YBK1, Y box binding protein 1.

### Biogenesis of circRNAs

circRNAs appear to be expressed in a tissue- and developmental stage-specific manner.^53^ circRNAs have been categorized into three types depending on their circularization mechanism: exonic, intronic, and retained-intron circRNAs. Exon-derived circRNAs are dominant among others such as 3′ untranslated region (UTR), 5′ UTR, introns, intergenic regions, and antisense RNAs.^54^ Exonic circRNAs account for more than 80% of known circRNAs, but their biogenesis mechanism is still unclear. It is known that complementary intron pairing is necessary for the inchoate back-splicing mechanism to take place.^55^ In 2003, two models were proposed for exonic circRNA formation: lariat-driven circularization and intron pairing-driven circularization.^56^ It is widely accepted that back-splicing occurs in a reversed orientation when a downstream 5′ splice site binds to an upstream 3′ splice site to circularize RNA in most instances.^57^ Intronic circRNA synthesis is different, as it depends on GU-rich sequences near the 5′ splice site and C-rich sequences near the branch point. The two segments bind to first form a circular structure. Afterward, specific spliceosomes facilitate the removal of exonic and intronic sequences at the binding sites. Finally, the remaining introns are joined together to form mature intronic circRNAs.^58^^,^^59^ Unlike the forward splicing of precursor (pre-)mRNAs, various circRNAs can be produced from a single gene locus by alternative back-splicing site selection.^57^^,^^60^ Despite the relatively low efficiency of back-splicing compared to forward-splicing of linear RNAs, there is still an abundance of circRNAs because of their stability and longer half-life.^61^

### Most recent discovery of differentially expressed circRNAs in cancer

The first step of investigating circRNAs in cancer is to identify which circRNAs are expressed differently than in the normal tissues. Galasso et al.^62^ examined a large group of circRNAs (n = 1,938) in breast cancer and described the genomic localization of circRNAs for the first time. They predicted that non-linear RNA would be resistant to RNase R treatment and confirmed the existence of circRNAs. With existing bioinformatics technologies, they developed a comprehensive workflow named “Circ-Seq” and applied it to 885 breast cancer samples. The results indicated that circRNA expression frequency may be a biomarker for oncogenesis in breast cancer.^63^ Zhang et al.^64^ also examined circRNAs in breast cancer. A total of 3,100 circRNAs were found to possess putative bind sites for members of the miR-200 family and let-7 family, which is relatively well characterized in breast cancer initiation and self-renewal. Although the main functions of circRNAs were thought to be acting as microRNA (miRNA) sponges and competing endogenous RNAs (ceRNAs), the enrichment of miRNA binding sites was not found to be a global feature, consistent with other recent studies.^6^^,^^64^^,^^65^ circRNAs possessing multiple miRNA-binding sites may not be highly expressed in cancer tissue.^56^ This study identified circRNA candidates for future functional characterization in breast cancer development.

Wan et al.^66^ reported that circITCH (circRNA itchy E3 ubiquitin protein ligase) was significantly downregulated in lung tumor tissues compared to paired adjacent non-cancerous tissues from 78 lung cancer patients. circITCH acted as oncogenic miR-7 and miR-214 sponges to enhance ITCH expression and suppress the activation of Wnt/β-catenin signaling. Thus, circITCH may inhibit lung cancer progression by enhancing its parental gene activities.^66^

circITCH was also downregulated in tissues of colorectal cancer (CRC).^54^ Similarly, Wang et al.^67^ revealed that hsa_circ_001988 was downregulated in colorectal tumor tissues. These results indicated that hsa_circ_001988 might be a biomarker of CRC. Bachmayr-Heyda et al.^68^ reported a global reduction of circRNA abundance in CRC cells both *in vitro* and *in vivo* compared to normal tissues and discovered a negative correlation between global circRNA abundance and tumor proliferation.

Esophageal squamous cell carcinoma (ESCC) is one of the most prevalent and fatal types of cancer worldwide, especially in Eastern Asia, and its prognosis remains poor. circITCH expression was downregulated in ESCC compared to peritumoral tissue. circITCH may play an important role in ESCC by regulating the Wnt/β-catenin pathway.^69^ hsa_circ_0067934 was significantly overexpressed in ESCC tissues compared to paired adjacent normal tissues and represented a novel potential biomarker and therapeutic target for ESCC.^70^ Additionally, circRNA_001059 and circRNA_000167 are implicated in esophageal cancer research. Su et al.^71^ performed a comprehensive screening of 3,752 candidate circRNA genes. Expression and functional profiles of the differentially expressed circRNAs in radioresistant esophageal cells revealed circRNA_001059 and circRNA_000167, which were the two largest nodes in the network through the circRNA-miRNA co-expression network.

Considerable amounts of circRNAs were detected in epithelial ovarian cancer (EOC) and were present in not only primary ovarian metastases but also peritoneal and lymph node metastases.^72^ Ahmed et al.^72^ constructed a regulatory network including circRNA and miRNA where multiple downstream effects may be activated by a single circRNA. The dysregulated circRNAs in ovarian cancers might be potential biomarkers, and the circRNAs that play an important role in carcinogenesis could be developed as therapeutic targets.

### Direct functional mechanisms of circRNA in tumor metastasis

Tumor metastasis is a detrimental component of cancer progression, and it poses a critical challenge in the treatment of many malignant tumors. The most common cause of death with malignant tumors is that cancer cells spread to other organs. Therefore, it is essential to uncover the molecular mechanisms of tumor metastasis. Non-coding RNAs, including circRNAs, have been shown to regulate metastasis in several types of cancers, and dysregulated circRNAs may lead to malignant behaviors. In recent years, an increasing number of circRNAs have been implicated in tumor metastasis. Some research groups have performed detailed investigations of circRNAs to understand the underlying mechanisms of their functions. circRNAs mainly influence tumor metastasis through miRNA sponging and protein-binding mechanisms.

#### circRNAs function as miRNA sponges

Most tumor-related circRNAs have been reported to function as miRNA sponges.^73^^,^^74^ Some miRNAs show abnormal expression in tumor tissue and could regulate its downstream signaling pathway to act as tumor suppressors or oncogenes, which could promote or inhibit the malignant behavior of tumors.^75^ ceRNAs are transcripts that can regulate each other at the post-transcriptional level by competing for shared miRNAs. circRNAs contain miRNA response elements (MREs),^76^ which enable them to act as ceRNAs to compete with their downstream target to regulate their downstream pathway. These circRNAs bind miRNAs to regulate their downstream targets, which may promote or suppress tumor metastasis.^77^, ^78^, ^79^

In urological tumors, Yang et al.^8^ found that circRGNEF was upregulated in bladder cancer (BC) cells, which enhanced their malignant behavior. They identified miR-548 and KIF2C (kinesin family member 2C) as downstream targets of circRGNEF. Silencing circRGNEF decreased invasion and migration of BC cells *in vitro* while downregulation of miR-548 and overexpression of KIF2C reversed these results. Also, Li et al.^9^ screened miRNAs sponged by upregulated circPRRC2A, and they found that miR-514a-5p and miR-6776-5p share common binding sites with circPRRC2A and TRPM3 (a protein that is highly related to malignant behavior of renal cell carcinoma [RCC]), indicating that circPRRC2A could regulate the DHX9-circPRRC2A-miR-514a-5p/miR-6776-5p-TRPM3 pathway in RCC through sponging miR-514a-5p, miR-6776-5p, miR-514a-5p, and miR-6776-5p, and they determined the regulatory role of the DHX9-circPRRC2A-miR-514a-5p/miR-6776-5p-TRPM3 pathway in RCC. Furthermore, Xue et al.^10^ observed downregulation of circAKT3 (circRNA AKT serine/threonine kinase 3) in clear cell RCC (ccRCC), resulting in the promotion of ccRCC migration and invasion. Bioinformatics and an RNA pull-down assay revealed that circAKT3 served as a sponge of miR-296-3p to upregulate E-cadherin, culminating in reduced migration and invasion of RCC. Li et al.^11^ studied overexpressed circTLK1 (circRNA tousled-like kinase 1) in RCC and discovered its positive correlation with distant metastasis. circTLK1 expression positively regulated CBX4 (chromobox 4) expression and ultimately increased VEGFA (vascular endothelial growth factor A) expression in RCC tissues.

In respiratory system tumors, Zhang et al.^12^ found that circFGFR1 (circRNA fibroblast growth factor receptor 1) was upregulated in non-small cell lung cancer (NSCLC) cells and served as a sponge for multiple miRNAs. Because of the specific enrichment of circFGFR1 and miR-381-3p, they screened miR-381-3p for further investigations. They concluded that circFGFR1 sponged miR-381-3p to upregulate CXCR4 (C-X-C motif chemokine receptor 4), which promoted the progression of NSCLC. Liu et al.^13^ showed that circRPMS1 (circRNA human gammaherpesvirus 4) encoded by Epstein-Barr virus (EBV) was upregulated in EBV-positive tissues and found that circRPMS1 could sponge miR-203, miR-31, and miR-451 to promote nasopharyngeal carcinoma (NPC) cell growth and epithelial-mesenchymal transition (EMT). In lung adenocarcinoma (LUAD) patients, Wang et al.^14^ studied a downregulated circRNA in LUAD tissues called circCRIM1 (circRNA cysteine-rich transmembrane BMP regulator 1). circCRIM1 was shown to inhibit LUAD cell migration and invasion both *in vitro* and *in vivo*. It was determined that circCRIM1 could sponge miR-182 and miR-93 to upregulate leukemia inhibitory factor receptor (LIFR) expression, a well-known tumor suppressor. By way of this pathway, circCRIM1 ultimately inhibited the invasion and metastasis of LUAD cells.

In gastrointestinal tumors, Lin et al.^15^ identified that circCYFIP2 (circRNA cytoplasmic FMR1 interacting protein 2) was upregulated in gastric cancer (GC) tissues and found that circCYFIP2 sponged miR-1205 and upregulated its downstream target gene E2F1 (E2F transcription factor 1), eventually promoting cell proliferation in GC cells. Chen et al.^16^ reported that circRUNX1 (circRNA RUNX family transcription factor 1) was upregulated in CRC and was associated with metastasis. Further studies revealed that circRUNX1 competitively sponged miR-145-5p, which is a tumor suppressor. Sponging miR-145-5p prevented its inhibitory effect on IGF1 (insulin-like growth factor 1), thereby promoting tumor metastasis. Wang and colleagues^17^ characterized circRNA transcripts from 10 GC and adjacent tissues by RNA-seq analysis. Among the 142 identified circRNAs, they conducted further studies on has_circ_0001725/circLMTK2 (circRNA lemur tyrosine kinase 2), which was derived from a protein-coding LMTK2 locus and upregulated in GC tissues. Silencing circLMTK2 significantly decreased cell proliferation, colony formation, and nucleotide synthesis. Additionally, they studied the function of circLMTK2 in cancer migration and metastasis. Results showed that knockdown of circLMTK2 eliminated the migratory and invasive capacities of AGS (human gastric adenocarcinoma) and MGC-803 cells *in vitro*. They validated these findings *in vivo* by injecting circLMTK2-overexpressed by lentivirus transfection (OE) and empty vector BGC-823 cells into mice and found significantly higher metastatic nodules in the circLMTK2-OE group. Mechanism studies revealed that circLMTK2 promoted metastasis by sponging miR-150-5p.^17^

In the genital system malignant tumor, Guo et al.^18^ showed that hsa_0023404 knockdown inhibited metastasis in cervical cancer (CC) cells. They further studied the mechanism of hsa_circ_0023404 in metastasis and showed that hsa_circ_0023404 acted as a sponge for miR-5047 to upregulate VEGFA, which was associated with the metastasis of cancer cells. Furthermore, hsa_circ_0023404 activated autophagy signaling to enhance chemoresistance. Lu et al.^19^ found that circEHMT1 (circRNA euchromatic histone-lysine-N-methyltransferases 1) was downregulated in human breast cancer cells. Overexpression of circEHMT1inhibited invasion and migration of human breast cancer cells. They discovered that miR-1233-3p was a target of circEHMT1 and showed that circEHMT1 could regulate MMP2 (matrix metallopeptidase 2), which is upregulated at sites of tissue damage, inflammation, and in stromal cells surrounding the invading front of metastatic tumors. Furthermore, miR-1233-3p could downregulate KLF4 (which belongs to the Krüppel family of transcription factors, has a dual function in cancer development, and acts as a tumor suppressor in breast cancer) at the mRNA and protein levels and reduce the MMP2 level indirectly through the circEHMT1/miR-1233-3p/KLF4 axis indirectly through the circEHMT1/miR-1233-3p/KLF4 axis to reduce MMP2 levels.

ciRS-7 is a circRNA transcribed from the CDR1 (cerebellum degeneration-related antigen 1) gene and is one of the earliest identified circRNAs and miRNA sponges in tumor cells.^7^ ciRS-7 binds miR-7 with high affinity, resulting in reduced miR-7 levels and increased levels of miR-7 targets. To understand the function of ciRS-7 in tumor progression, the regulatory network of miR-7 needs to be discussed. miR-7 is a negative regulator of degradation of α-synuclein and an inhibitor of pancreatic β cells induced mTOR signaling with effects on cell proliferation.^7^^,^^80^^,^^81^ miR-7 downregulates central oncogenic factors in cancer-related pathways such as EGF (epidermal growth factor) receptor, IRS-1 (insulin receptor substrate-1), IRS-2 (insulin receptor substrate-2), Pak1 (p21-activated kinase 1), and PIK3CD (phosphoinositide-3-kinase, catalytic, delta polypeptide), demonstrating that miR-7 exerts an important tumor-suppressive role via widespread regulatory control.^7^ Hu et al.^25^ identified a circRNA, circASAP (circRNA microtubule-associated protein 9), that was associated with pulmonary metastasis after curative resection in hepatocellular carcinoma patients. They found that circASAP1 promoted cell proliferation, migration, and invasion *in vitro*. Unsurprisingly, circASAP1 promoted tumor growth and pulmonary metastasis *in vivo*. These functions were carried out by acting as a ceRNA for known tumor suppressors in hepatocellular carcinoma, i.e., miR-326 and miR-532-5p, and subsequently regulating MAPK1 (mitogen-activated protein kinase 1) and CSF-1 (colony-stimulating factor 1) pathways.^25^ ciRS-7 is also known to sponge miR-671, and miR-7 can be released when ciRS-7 binds miR-671. Additionally, ciRS-7 regulates the suppression of target oncogenes and promotes metastasis, vascularization, and the proliferation of tumor cells.^82^ ciRS-7 and circular Sry are the best characterized miRNA sponges containing multiple miRNA binding sites to date.^83^ All of this research reveals that ciRS-7 can sponge different miRNAs to regulate downstream targets and influence tumor progression, which indicates that miRNA sponges may represent major and extensive mechanisms of circRNAs in tumor progression. Several reports suggest that most circRNAs likely do not function as miRNA sponges.^84^ However, most circRNAs being linked to cancer development are reported to function as miRNA sponges. Elucidating the complex downstream pathways and regulatory networks will allow us to further understand the roles of circRNAs in cancer development. circRNAs could be potential cancer biomarker candidates and therapeutic targets for cancers.^85^

#### circRNAs binding to protein

circRNAs also interact with proteins to activate or inhibit downstream signaling pathways to affect the malignant behavior of tumors.^86^ Expression of circFoxo3 (circRNA forkhead box O3) suppressed cell proliferation and cell cycle progression because circFoxo3 formed a ternary complex with CDK2 (cyclin-dependent kinase 2) and p21 to inhibit the formation of cyclin E/CDK2 and cyclin A/CDK2 complexes, thereby arresting cell cycle progression at the G_1_ phase^6^. Upregulation of circFoxo3 was found to induce cell apoptosis by binding to MDM2 (murine double minute-2) and preventing MDM2-mediated ubiquitination of Foxo3.^87^ Chen et al.^77^ found that circAGO2 (circRNA Argonaute 2) was upregulated in several types of human cancer tissues and promoted the growth, invasion, and metastasis of cancer cells. circAGO2 interacted with and activated HuR (human antigen R) protein in cancer cells, which inhibited downstream miRNAs and facilitated cancer progression. These results indicate that circAGO2 and HuR can be therapeutic targets for many types of cancers.

In digestive system tumors, Jiang et al.^20^ found that circ_0032821 was upregulated in human GC tissue and cells and could positively modulate the MEK1/ERK1/2 signaling pathway to promote tumor malignancy. Hu et al.^21^ found that circGSK3β (circRNA glycogen synthase kinase-3β) was overexpressed in ESCC tissues and played a role in ESCC cell metastasis. Pull-down assays revealed GSK3β as a downstream target of circGSK3β. Furthermore, the GSK3β/β-catenin signaling pathway was demonstrated to promote ESCC metastasis. They also tested the plasma concentration of circGSK3β in ESCC patients and concluded that plasma circGSK3β could be a potential biomarker for ESCC diagnosis and prognosis. In addition, Chen et al.^22^ found that circNSUN2 (circRNA NOP2 RNA methyltransferase 2) was highly expressed in liver metastasis (LM) tissues from CRC patients. It was demonstrated that circNSUN2 enhanced the mRNA stability of HMGA2 (high mobility group AT-hook 2) through the formation of a circNSUN2/IGF2BP2/HMGA2 RNA-protein ternary complex, ultimately promoting the LM of CRC. Yang et al.^23^ found that circHuR was downregulated in GC cells. It suppressed the transcription of HuR by inhibiting CNBP (CCHC-type zinc-finger nucleic acid binding protein) transactivation through interaction with its RGG domain. CNBP overexpression promoted lung metastasis of GC *in vivo*. Thus, circHuR played a tumor-suppressor role in GC. It can suppress the transcriptional activity of CNBP, and it functions as an endogenous inhibitor for repressing the binding of CNBP to the HuR promoter, resulting in the downregulation of HuR and its target genes involved in cancer progression. Wong et al.^24^ identified circFOXK2 as an upregulated circRNA in pancreatic ductal adenocarcinoma (PDAC) and confirmed that circFOXK2 (circRNA forkhead box K2) promotes tumor growth and metastasis *in vivo*. They then used mass spectrometry and immunoprecipitation assays to identify circFOXK2 protein interactions and uncovered its interaction with YBX1 (Y box binding protein 1) and hnRNPK (heterogeneous nuclear ribonucleoprotein K) proteins, increasing binding of YBX1 and hnRNPK to NUF2 (nuclear division cycle 80 kinetochore complex component) and PDXK (pyridoxal kinase), in turn increasing their expression. These results showed that circFOXK2 promoted the expression of oncogenic proteins NUF2 and PDXK, which contribute to the progression of PDAC, revealing a novel target for diagnosis and treatment of PDAC. In breast cancer tissue, circCCNB1 (circRNA cyclin B1) formed a complex with H2AX (histone family 2A variant) and Bclaf1 (B cell leukemia/lymphoma 2 apoptosis regulator-associated transcription factor 1) to induce cell death in p53 mutant cells.^26^ This circRNA could also inhibit the malignant behaviors of cancer cells by forming a complex with CCNB1 and Cdk1 (cyclin-dependent kinase 1) to suppress the oncogenic functions of CCNB1.^27^ In contrast to circCCNB1, circSKA3 (circRNA spindle and kinetochore-associated complex subunit 3) was upregulated in human breast cancer, where it enhances cell migration and invasion.^28^ This circRNA promotes invadopodium formation through binding and bridging Itgb1 (integrin subunit beta 1) and Tks5 (sperm hammerhead 3 and protamine 3 domains 2A).^28^ Zheng et al.^29^ found that the upregulation of circSEPT9 (circRNA septin 9) was mediated by E2F1, and EIF4A3 (eukaryotic translation initiation factor 4A3).circSEPT9 subsequently sponged miR-637 and activated the LIF/Stat3 signaling pathway to promote the progression of triple-negative breast cancer (TNBC).

In summary, circRNAs could interact with multiple proteins to regulate the function or translation of them or their downstream targets to influence tumor metastasis. All of these findings suggest that circRNA protein interactions could be an appropriate target in tumor metastasis.

#### circRNAs regulating their host gene

Some circRNAs have been reported to regulate their host gene to affect tumor metastasis. circRNAs are generated through exon skipping or back-splicing from their host gene. In some cancer tissue, circRNAs could affect their host gene through mechanisms such as miRNA sponging or protein binding to regulate their downstream pathway and regulate the expression of their host genes, which have a positive or negative impact on tumor metastasis. Zhou et al.^30^ found that circENO1 (circRNA enolase 1) was upregulated in LUAD cells. The binding sites on circENO1 specific to miR-22-3p were identified. They confirmed that circENO1 could sponge miR-22-3p to upregulate its host gene ENO1, which promoted glycolysis and led to malignant behaviors in LUAD. They also proved that circENO1 promoted tumor growth and metastasis in LUAD *in vivo*. These results indicate that circENO1 could be a novel potential biomarker in lung cancer. In contrast, Li et al.^31^ found that both circITGA7 (circRNA integrin subunit alpha 7) and its host gene ITGA7 could inhibit CRC cell proliferation and metastasis. The transcription of ITGA7 was upregulated by circITGA7 through the suppression of RREB1 (Ras responsive element binding protein 1) via the Ras pathway. YAP (yes-associated protein) was negatively regulated by circYAP through the suppression of the assembly of Yap translation regulators such as eIF4G (eukaryotic translation initiation factor 4 gamma) and PABP (poly(A)-binding protein).^32^ This circRNA is dysregulated in breast cancer tissues and may function as a potential target for future breast cancer intervention. All of the above results show that circRNA can act as a factor to affect some signaling pathways to regulate their host gene indirectly. The host gene should also be considered as a target to discover the molecular mechanism of tumor metastasis. However, there is limited evidence of circRNAs regulating their host gene directly or indirectly. Future research should further investigate this circRNA mechanisms in tumor metastasis.

#### circRNA translation into protein

The biological functions of most circRNAs remain unclear. Although hundreds of endogenous circRNAs have been reported to have translation potential, only a few circRNA translation products have actually been detected.^88^, ^89^, ^90^ circRNA translation products may play important roles in tumor progression.

circZNF609 was selected from a high-content functional genomic screening and has a function in muscle differentiation and myoblast proliferation.^89^ A start codon and an in-frame stop codon were located in circZNF609, which suggested that it has the potential to be translated. It was confirmed that circZNF609 is associated with many polysomes and can be translated into a protein in a splicing-dependent and cap-independent fashion. circZNF609 originated from the second exon of its host gene, a 753-nt open reading frame (ORF) from the AUG of the host gene to a stop codon that is 3 nt after the splice junction.^89^

The function of the protein derived from circZNF609 has not yet been elucidated. There is also limited information about the zinc-finger protein 609 protein activity. As one of the largest transcription factor families, zinc-finger proteins can have versatile functions in biological processes due to the diverse combinations of zinc-finger motifs. Their involvement in development, differentiation, metabolism, and autophagy makes zinc-finger proteins important candidates in cancer studies. Preliminary studies have shown the potential role of zinc-finger proteins in cancer progression. The underlying mechanisms of these proteins are still unclear, as there appear to be various mechanisms even within the same cancer.^91^ The circZNF609-encoded protein lacks the zinc-finger domain, suggesting that circZNF609 could potentially act as a dominant-negative competitor to regulate cancer progression.^89^ Cap-independent translation was driven by IRES (internal ribosome entry site). This mechanism was first described in viral RNAs^92^ and later identified in cellular RNAs.^93^ IRES elements can act via many mechanisms, such as forming RNA structures and cooperating with IRES-transacting factors.^94^ Putative cellular IRES elements have been identified to play crucial roles in different developmental conditions and response to different types of stress.^95^ Considering the IRES-dependent translation of circZNF609, the splicing event might not only play crucial roles in ribosome recognition and translation initiation but also in tumor progression or cancer cell apoptosis.^89^ This may be an area of investigation in future studies.

Another circRNA translation study was conducted by Pamudurti et al.,^90^ who reported that a circRNA generated from the muscle-blind locus encodes a protein, detected in fly head extracts using ribosome footprinting datasets and searching for reads across the circRNA-specific junctions.^96^^,^^97^ By performing *in vivo* and *in vitro* translation assays, they found that UTRs of ribo-circRNAs (cUTRs) induced cap-independent translation.^90^ The translation abilities of ribo-circRNAs,circMbl (circRNA mannose-binding lectin), circCdi (circRNA center divider), and circPde8 (circRNA phosphodiesterase 8) were identified, and the minigenes were engineered to expressed V5-tagged protein in case of a circularization event. It was found that the minigene-derived circRNA molecules were associated with translating ribosomes and consistently detected MBL-immunoreactive bands, strongly suggesting that circMbl can be translated into a protein. The *mbl* locus produced several circRNAs, some of which were highly abundant. They also detected a circMbl3-derived 37.04-kDa protein.^90^

It was suggested that circRNAs could be selectively translated from the same linear RNA, and that the background mechanism of circRNA translation could affect tumor biogenesis in an extremely complicated manner. Although the circRNA is translated in a cap-independent manner, the mechanism is not used by linear mRNA. The circRNA-derived proteins were identified but are yet to be clearly linked to the tumor. More findings might be available when more proteins are identified and studied in cancer diseases.

### Indirect circRNA functions in tumor metastasis

Some biological effects show a high correlation with tumor metastasis. The process of these biological effects usually has circRNA participation and affects such biological effects.

#### Exosomal circRNAs

Exosomes are types of nano-sized vesicles (30–150 nm) that carry biomolecules, including proteins, glycans, lipids, metabolites, RNA, and DNA, that are secreted by most cells. Exosomes and their contents can be absorbed by other cells to influence their phenotypes.^98^ In recent years, several findings regarding exosomal circRNAs have been reported, providing a new potential approach to malignant tumor therapy.

Huang et al.^33^ found that exosomal circRNA-100,338 was highly expressed in metastatic HCC cells and their secreted exosomes. Overexpression of circRNA-100,338 was associated with pulmonary metastasis of HCC. These findings first showed the potential function of exosomal circRNAs in cancer progression and provided a theoretical basis for follow-up studies. Zhao et al.^34^ proved that exosomal circABCC1 (circRNA ATP binding cassette subfamily C member 1) secreted from CD133^+^ cells, isolated from CRC cells, could mediate cell stemness and metastasis through the WNT pathway. They concluded that circABCC1 could be a potential therapeutic agent for CRC. Furthermore, Li et al.^35^ observed upregulated circ_0044516 in exosomes in prostate cancer cell lines. A later study found that circ_0044516 could sponge miR-29a-3p, a tumor-suppressing miRNA, to enhance prostate cancer cell metastasis. These effects could be rescued by inhibiting circ_0044516, providing a novel therapeutic target for prostate cancer. Liu et al.^36^ found that circ_MMP2 was upregulated in metastatic HCC tissues and was delivered by 97H- or LM3-secreted exosomes. It functioned as a ceRNA to sequester miR-136-5p and regulate its metastasis-related host gene MMP2a protein, ultimately promoting tumor metastasis. Alternatively, Chen et al.^37^ discovered an exosomal circRNA hsa_circ_0051443 released by normal cells that were downregulated in HCC tissues and plasma. Exosomes containing hsa_circ_0051443 were delivered to HCC cells to inhibit proliferation and migration through sponging miR-331-3p to protect its target gene BAK1, which is associated with the occurrence of multiple tumors. This phenomenon is observed *in vitro* and *in vivo* and indicates that hsa_circ_0051443 could be a potential biomarker and therapeutic target for HCC. Exosomes are thought to be involved in intercellular communication. Exosomal circRNAs in particular have been implicated in cancer development. In summary, exosomal circRNAs are a series of circRNAs carried by exosomes that could interact cell to cell and influence the malignant behaviors of tumor cells. The findings above provide a theoretical basis for further research.

#### circRNAs regulate EMT

Although the functional mechanisms discussed in the previous sections have been more extensively studied, other functions of circRNAs in tumor metastasis are also noteworthy. The EMT is an evolutionarily conserved process in which epithelial cells lose their intercellular adhesion and polarity, which exacerbates their invasive capacity. Although EMT is critical for dramatic cellular movements during embryogenesis, tumor cells can reactivate EMT programs, which increases their aggressiveness. EMT is associated with enhanced stem cell properties and drug resistance, which can drive metastasis, tumor recurrence, and therapy resistance in the context of cancer.^75^ EMT confers metastatic properties in cancer cells by enhancing migration, invasion, and resistance to apoptosis (the process of programmed cell death).^99^

circRNAs potentially affect tumor metastasis by activating the EMT process. Yan et al.^38^ used high-throughput sequencing to identify differences in circRNA and miRNA expression in prostate cancer cells undergoing EMT. Following this, hsa_circ_0001165 and hsa_circ_0001085 were screened to determine their potential signaling pathways. They found that hsa_circ_0001165 regulated TNF (tumor necrosis factor) expression through miR-187-3p to induce EMT in prostate cancer cells. Potentially, hsa_circ_0001085 also induced EMT by sponging miR-196b-5p and miR-451a through the TGF-β (transforming growth factor β) and MAPK signaling pathways. Similarly, Yuan et al.^39^ discovered EMT-related circRNA expression profiles in breast cancer cells and subsequently screened circSCYL2 (circRNA SCY1-like pseudokinase 2) and its target gene OSR1 (odd-skipped related transcription factor 1), which is a suppressor of malignant behaviors of GC and RCC. Both were downregulated in breast cancer. These findings suggest circSCYL2 and OSR1 could be therapeutic targets to inhibit EMT in breast cancer. Furthermore, Zhou et al.^40^ found that circRNA_0023642 was upregulated in GC tissues and cell lines compared to adjacent normal tissues. Silencing circRNA_0023642 inhibited HCG-27 (human gastric carcinoma cell line 27) cell migration and invasion *in vitro*. They further studied EMT-related genes and found downregulation of N-cadherin, vimentin, and snail and upregulation of E-cadherin at both mRNA and protein levels, which were reversed compared to normal tissues undergoing EMT, indicating that silencing circRNA_0023642 may inhibit tumor metastasis through inhibiting the EMT process. In addition, Qian et al.^41^ reported that EMT was positively regulated by circHIPK3 (circRNA homeodomain interacting protein kinase 3) in CC cells. circHIPK3 sponged miR-338-3p to upregulate its downstream target HIF-1α (hypoxia-inducible factor-1α) and promote EMT. These effects could be rescued by knocking down circHIPK3. Another study conducted by Wang et al.^42^ investigated the relationship between circPTK2 (circRNA protein tyrosine kinase 2) and EMT in NSCLC. Overexpressed circPTK2 protected TIF1γ (transcriptional intermediary factor 1 gamma), an essential regulator of TGF-β signaling and EMT, by sponging miR-429/miR-200b-3p in NSCLC cells, culminating in the inhibition of EMT and tumor metastasis. Wei et al.^43^ also found that circPTPRA (circRNA protein tyrosine phosphatase receptor type A) acted as a miRNA sponge in NSCLC for oncogenic miR-96-5p, suppressing EMT through the miR-96-5p/RASSF8/E-cadherin axis. Lastly, Pan et al.^44^ revealed that overexpression of hsa_circ_0006948 in ESCC could induce EMT to promote ESCC progression. This effect was mediated by sponging miR-490-3p and enhancing HMGA2 expression. miRNA sponging has been an extensively reported circRNA mechanism in EMT, suggesting that it may be a core mechanism of circRNA implicated in EMT.

#### circRNAs regulate autophagy

Autophagy refers to the process in which cells phagocytose proteins and organelles using autophagosomal vesicles and deliver them to the lysosome for degradation. This process plays an essential role in the metabolism of cells and the renewal of some organelles, and its dysfunction is associated with many human diseases.^100^ Much evidence suggests that autophagy could be a potential therapeutic target in cancer.^101^ A few circRNAs have been implicated in autophagy and tumor metastasis. The circRNA circDNMT1 (circRNA itchy E3 ubiquitin protein ligase) could be upregulated in breast cancer cell lines and patient tumor samples. circDNMT1 interacted with p53 and AUF1 (AU-rich element-binding factor 1) proteins to promote their nuclear translocation. Nuclear translocation of AUF1 reduced the instability of DNMT1 mRNA to inhibit the transcription of p53, enhancing cellular autophagy in breast cancer.^45^ Gan et al.^46^ discovered that circMUC16 (circRNA mucin 16, cell surface associated) was upregulated in EOC and promoted autophagy, thereby contributing to EOC invasion and metastasis. Their findings indicate that circMUC16 is a potential target for the diagnosis and treatment of ovarian cancer. Autophagy plays a key role in cancer processes and metastasis, and we need further study to discover the function of circRNAs in this cellular metabolism process.

## Conclusions and future perspectives

There has been increasing interest in the physiological and pathological functional mechanisms of circRNAs.^7^^,^^102^^,^^103^ Although circRNAs were first discovered around 40 years ago, most circRNA research has been done in recent years. The discoveries of circRNA roles in apoptosis, oxidative stress, heat shock response, pathogen infection, and especially in tumor progression have made steady progress but still require further research. The recent introduction of genome-editing techniques such as CRISPR-Cas9,^104^ which can induce a single-site mutation at splice sites or introns that are involved in circularization, can be used to further investigate the physiological significance of these circRNAs in tumor biology.

Given the relatively low abundance of most circRNAs, the quantitative threshold for these small molecules to carry out significant functions in tumor biology should be determined. As the disease might be affected by a complex network of circRNAs functioning instead of a single circRNA, the logical research progress would be to screen the circRNAs and then study the function of a group of circRNAs or single circRNAs that are significantly differentiated. Future studies on circRNAs and their potential targets are needed to uncover more roles of circRNAs in tumor progression, as the numbers of findings on miRNA sponging circRNAs do not necessarily mean that most circRNAs indeed function that way.

Today, circRNAs are not considered “non-coding,” as endogenous circRNAs are associated with ribosomes with RRACH sequences.^88^ IRES- or RRACH-induced circRNA translation may have important functions for tumor progression under different conditions when cap-dependent translation is not possible. It is well known that cap-independent initiation is less efficient.^105^ However, the regulation of cap-independent translation is often used to immediately respond to cellular stresses by inducing selective changes in protein levels. These changes may be responsible for early cancer cell biogenesis, and different translation modes between linear mRNA and circRNA may have important effects on the regulation of cancer cell proliferation, migration, and survival. Extensive research has confirmed the functional significance and mechanisms of circRNAs in tumor metastasis. Future studies should aim to develop novel methods to treat these various types of cancer.

There is a competition between circRNA biogenesis and the forward-splicing of their linear counterparts,^106^ and splicing factors or splicing events may have functions in circRNA biogenesis during tumor progression. Splicing events are associated with the intron size and splicing factor, and splicing patterns can be altered under different physiological conditions. The resulting circRNAs may have more important functions in tumor biology compared to their linear counterparts. Therefore, it is critical to understand the mechanisms by which splicing regulates circRNA biogenesis during tumor progression.
